# Viral Encephalitis in England, 1989–1998: What Did We Miss?

**DOI:** 10.3201/eid0902.020218

**Published:** 2003-02

**Authors:** Katy L. Davison, Natasha S. Crowcroft, Mary E. Ramsay, David W.G. Brown, Nick J Andrews

**Affiliations:** * Public Health Laboratory Service Communicable Disease Surveillance Centre, London, United Kingdom; † Public Health Laboratory Service Virus Reference Division, London, United Kingdom

**Keywords:** Viral encephalitis, herpes simplex encephalitis, surveillance, etiology, epidemiology, outbreaks, research

## Abstract

We analyzed hospitalizations in England from April 1, 1989, to March 31, 1998, and identified approximately 700 cases, 46 fatal, from viral encephalitis that occurred during each year; most (60%) were of unknown etiology. Of cases with a diagnosis, the largest proportion was herpes simplex encephalitis. Using normal and Poisson regression, we identified six possible clusters of unknown etiology. Over 75% of hospitalizations are not reported through the routine laboratory and clinical notification systems, resulting in underdiagnosis of viral encephalitis in England. Current surveillance greatly underascertains incidence of the disease and existence of clusters; in general, outbreaks are undetected. Surveillance systems must be adapted to detect major changes in epidemiology so that timely control measures can be implemented.

Routine laboratory reports and statutory clinical notifications of infectious diseases are a source of viral encephalitis surveillance in England; however, these methods are considered to be incomplete. Hospital episode statistics record hospital admissions of viral encephalitis; although not timely enough for surveillance, these statistics can be used to monitor the distribution of admissions attributed to viral encephalitis by hospital physicians.

Most cases of viral encephalitis in the U.K. are thought to be rare complications of common infections. Herpes simplex virus (hereafter referred to as herpes) is the virus most often associated with encephalitis (*1*–*3*). Other viruses known to cause encephalitis include varicella-zoster virus 1 (VZV), measles virus, rubella virus, Lymphocytic choriomeningitis virus, cytomegalovirus, Epstein-Barr virus, and the adenoviruses (*4*–*6*). Cases of encephalitis attributed to arthropod-borne viruses (arboviruses) are common in certain areas of the world, but only rare, imported cases have been reported in the U.K (*7*,*8*).

Clinical diagnosis of viral encephalitis is based on symptoms such as fever, headache, and altered mental state; however, a definitive diagnosis of viral encephalitis relies on detecting the virus in cerebrospinal fluid or brain. The use of virologic investigation has been inconsistent in England. Virus isolation, formerly the standard criterion for diagnosis, is steadily being replaced by polymerase chain reaction (PCR) (*9*–*11*), which is more sensitive and provides a more rapid result. However, PCR was not widely used at the time of this study (*9*,*12*,*13*).

The demonstration of specific intrathecal antibody, either by detecting a raised antibody index or by detecting specific oligoclonal bands in cerebrospinal fluid, provides evidence of recent neurologic infection. When combined with an appropriate clinical picture, this demonstration is considered diagnostic (*14*). A noninvasive investigation such as magnetic resonance imaging (MRI) is also diagnostic (*15*). Serologic confirmation based on a fourfold rise in the level of antibody to the virus in the acute- and convalescent-phase blood samples or the demonstration of an intrathecal antibody response also provides evidence of recent infection.

The accurate diagnosis of viral encephalitis is important, particularly so for herpes and VZV encephalitis because several effective antiviral drugs are widely available for treatment (*1*,*16*,*17*). Accurate diagnosis is also required to increase the usefulness of surveillance so that the pattern of viral encephalitis cases can be monitored, especially since concern is increasing about the potential problems posed by new and reemerging infections (*18*). As new treatments and vaccines for existing viral infections become available, good surveillance data are required to accurately describe the public health cost of viral encephalitis, develop appropriate vaccination strategies, and perform treatment algorithms (available from: URL: http://www.isabel.org.uk/) (*19*,*20*).

This study describes the epidemiology of viral encephalitis in England from 1989 to 1998 by using hospital episode statistics (available from: URL: http://www.doh.gov.uk/hes/) and evaluates routine surveillance systems in terms of their ability to quickly and accurately monitor sporadic cases and clusters. We also consider possible future surveillance methods.

## Methods

### Data Sources

Hospital episode statistics provide information on hospital care in National Health System hospitals in England. No case definition is available for viral encephalitis, but diagnosis is generally based upon clinical evidence of viral encephalitis and available confirmatory laboratory data as recorded in the patient’s medical record at the time of discharge. The diagnosis is recorded by using the World Health Organization International Classification of Disease (ICD) codes. These codes allow for specific (clinical and laboratory evidence) and nonspecific (only clinical evidence) diagnoses. We analyzed reports of hospital admissions for all adults and children (<17 years of age) from April 1, 1989, to March 31, 1998. Cases of viral encephalitis were identified by ICD ninth edition (ICD-9) codes for admissions in 1989–1995 and 10th edition (ICD-10) codes for admissions in 1995–1998. Patient records were also extracted if any of seven diagnostic codes mentioned a diagnosis of viral encephalitis (Table 1). Since no ICD-9 code has been defined for VZV or adenovirus encephalitis, any VZV or adenovirus infection code accompanied by a code for nonspecific viral encephalitis before 1995 was included in the analysis. Multiple episodes for one patient were considered to be a single infection if <1 month elapsed between episodes.

**Table 1:** Diagnostic codes used to identify cases of viral encephalitis in hospital episode statistics

Encephalitis diagnosis			ICD-9^a^	ICD-10^a^
Specific diagnosis:	Exotic virus		0620–0629, 0630–0638, 064, 0661, 0622, 3233	A830–A839, A840–A849, A852
Herpes simplex virus			0543	B004
Varicella-zoster virus 1			Undefined	B011, B020
Measles virus			0550	B050
Mumps			0722	B262
Rubella			0560, 0567	B060
Lymphocytic choriomeningitis virus			0490, 3126	A872
Adenovirus			Undefined	A851
Other			0498, 3234	A858
Nonspecific diagnosis:		Unspecified	0499, 3239	A86, G051

^a^ICD, International Classification of Disease.

The second data source was laboratory reports sent to the Communicable Disease Surveillance Centre from the Public Health Laboratory Service (PHLS), National Health System, and private laboratories in England. All laboratory reports of isolates of viruses known to cause encephalitis from January 1, 1990, to December 31, 1998, were extracted from LabBase (the PHLS laboratory network electronic database). From these reports, infections thought to be associated with encephalitis were identified. Data extracted included laboratory reports in England with specimen date between January 1, 1990, and December 31, 1998, of adenoviruses, herpes, VZV, cytomegalovirus, lymphocytic choriomeningitis virus, Epstein-Barr virus, measles, mumps, or rubella detected in the cerebrospinal fluid, or adenovirus, herpes, VZV, cytomegalovirus, lymphocytic choriomeningitis virus, Epstein-Barr virus, measles, mumps, or rubella in any specimen with an encephalitis diagnosis in the feature or comment field.

An additional data source was the Notifications of Infectious Disease System (NOIDS) (available from: URL: www.phls.co.uk/facts/NOIDS/noid.htm). Clinically diagnosed cases of viral encephalitis are reported weekly as acute, infectious encephalitis to consultants in Communicable Disease Control and collated by the Communicable Disease Surveillance Centre. Data collected included the week of the report and patients’ age, sex, and local health authority of residence. Reports from 1989 through 1998 were available for analysis.

The final source was certified deaths reported to the Office of National Statistics. Information about deaths attributed to viral encephalitis from January 1, 1993, to December 31, 1998, in England was available from Office of National Statistics data held by the Communicable Disease Surveillance Centre. All deaths were coded with an ICD-9 designation.

### Analysis

We investigated trends in hospitalizations of viral encephalitis by fiscal year, using 1995 mid-year resident population estimates for England to calculate rates. Single- and multi-variable regression was used to compare rates by fiscal year, age group (<1, 1–4, 5–9, 10–16, 17–24, 25–34, 35–44, 45–54, 55–64, and >65 years of age), sex, and regional health authority of hospital (Anglia and Oxford, North West, South and West, Trent, North Thames, Northern and Yorkshire, South Thames, and West Midlands). We initially investigated evidence of clustering of case-patients with a nonspecific diagnosis by comparing weekly totals of hospitalizations of nonspecific viral encephalitis to the weekly overall mean number during the study period. Possible clusters were considered to have occurred when the weekly total was >2.58 and >3.3 standard deviations from the mean, corresponding to 99.5% and 99.95% upper prediction limits, respectively. In addition, we used Poisson regression to calculate an upper weekly and monthly prediction limit (at 99.5% and 99.95%) for each hospital’s district health authority. Any weekly or monthly count greater than or equal to the limit in each district was flagged as a possible outbreak. We compared NOIDS and laboratory reports to hospital episode statistics to determine the level of underreporting in each.

## Results

### Hospital Episode Statistics

From April 1, 1989, to March 31, 1998, a total of 6,414 adults and children were hospitalized in England with a diagnosis of viral encephalitis (Table 2), a figure that corresponds to an estimated annual rate of 1.5 cases per 100,000 population. Most of these patients had a nonspecific infection. A specific diagnosis was recorded for 2,574 patients, the largest proportion of which (52%) had herpes infection. The other diagnoses included 18% with “other” and 13% with VZV. The remaining 223 included patients with encephalitis associated with measles, mumps, rubella, exotic viruses (arboviruses), and Lymphocytic choriomeningitis virus infections.

**Table 2:** Hospitalizations of viral encephalitis by diagnosis and fiscal year of admission, April 1, 1989–March 31, 1998, England

Diagnosis	Date of admission by fiscal yr									
	1989–90	1990–91	1991–92	1992–93	1993–94	1994–95	1995–96	1996–97	1997–98	Total
Exotic	3	3	7	7	5	9	20	7	3	64
Herpes viruses										
Herpes simplex^a^	138	163	170	175	172	168	120	147	166	1,419
Varicella zoster	8	17	13	8	8	6	94	99	80	333
Others										
Measles	16	8	8	7	8	23	7	5	4	86
Mumps	12	5	2	3	1	0	2	3	2	30
Rubella	5	3	3	1	2	5	5	1	1	26
LCMV^b^	0	0	2	0	0	0	0	2	3	7
Adenoviruses	19	15	21	14	17	14	12	10	7	129
Other^c^	47	75	56	51	62	48	58	46	37	480
Total (%)	248 (40)	289 (40)	282 (37)	266 (37)	275 (33)	273 (36)	318 (47)	320 (48)	303 (47)	2,574 (40)
Unspecified viral infection (%)	379 (60)	422 (60)	488 (63)	461 (63)	551 (67)	480 (64)	365 (53)	351 (52)	343 (53)	3,840 (60)
Total (%)	627 (100)	711 (100)	770 (100)	727 (100)	826 (100)	753 (100)	683 (100)	671 (100)	646 (100)	6,414 (100)

^a^Herpes simplex virus is undefined after 1995–1996 and recorded thereafter as herpes.

^b^LCMV, Lymphocytic choriomeningitis virus.

^c^Both “other” and “unspecified” are included because both groups existed from 1995 to

1996 onward.

A total of 2,734 cases were diagnosed in children (including 35 in neonates), corresponding to an estimated annual rate of 2.8 per 100 000 children and accounting for 43% of all hospitalizations of viral encephalitis. An estimated 21% declining trend occurred in the rate over the 9 years, although this decline is not significant (p=0.065). Over half (51%) of the hospitalizations of children occurred for those <5 years of age, with the highest rate (8.7/100 000) for infants <1 year of age. The rate remained highest for infants throughout the 9 years of the study; however, we found some variation between the age groups in the time trends that approached significance (p=0.063), with the largest decline in adolescents ages 10–16 years (estimated to be 37%) and the smallest in toddlers 1–4 years of age (estimated to be 4%). Little difference appeared in the rate in male children compared to female children overall (2.9 and 2.8/100,000 children; p=0.23); furthermore, the trend over the 9 years did not differ by sex (p=0.63).

In adults, 3,680 patients diagnosed with encephalitis were hospitalized, which approximates an annual rate of 1.1 per 100 000 adults with no overall significant trend over time (p=0.17). No significant variation appeared in the rate between the age groups overall, but the highest rate occurred in those aged 17–24 years at approximately 1.2 per 100,000. The change in the incidence of hospitalized case-patients over time varied between age groups (p=0.0016); this change was mostly attributable to an increase of 46% in the rate in elderly case-patients (>65 years of age) with no significant changes in other age groups. We found no difference in the overall rate of male case-patients compared to female case-patients (1.1 and 1.0/100,000 adults; p=0.12); furthermore, the trend over the 9 years did not differ by sex (p=0.74).

The rate between the regional health authority of hospitals did not vary significantly overall (p=0.55); however, the largest proportion of cases (16%) and highest rate (1.63/100 000 population) were hospitalized in the North Thames region. The trend over the 9 years did not significantly vary by regional health authority (p=0.13).

The proportion of cases with a specific diagnosis was significantly lower in children (33%) than adults (45%) (p<0.001). In children, this proportion varied throughout the study (p<0.001); it was at a minimum of 23% during 1992–1993 and a maximum of 50% during 1996–1997. Children with a specific diagnosis were significantly younger than those without (mean age of 5 vs. 6.1 years; p<0.001) but did not differ in terms of sex. In adults, the proportion with a specific diagnosis did not vary significantly by year. Those with a specific diagnosis were significantly older compared to those without (mean age 49 vs. 43 years; p<0.001) but did not differ in terms of sex. The proportion of all cases with a specific diagnosis varied significantly by region, ranging from 34% in Trent to 47% in Northern and Yorkshire (p<0.001). The regions of Northern and Yorkshire had the highest proportion of herpes encephalitis (28.35%); in comparison, Trent had 21.4%, and North West (the region with the lowest proportion) had 18%.

In hospitalized case-patients, 417 deaths were identified during the 9 years of study. The severity of cases was compared by using a crude case-fatality rate and mean length of stay (Table 3). The overall case-fatality rate was 6.5 per 100 cases, and the mean length of stay was 17.5 days. Considerable variation appeared in case-fatality rate and mean length of stay among the different diagnoses. Herpes encephalitis had the highest overall case-fatality rate and mean length of stay and was considered to be the most severe diagnosis. In children, the overall case-fatality rate was 2.3 per 100 cases (95% confidence interval [CI] 1.8 to 3), 1.9 per 100 cases of herpes (95% CI 0.7 to 4), and 2.1 per 100 cases of viral encephalitis without a specific diagnosis (95% CI 1.5 to 2.8). The mean length of stay for children was 10.9 days, increasing to 11.6 days for patients without a diagnosis specified and 13.2 days for patients with herpes encephalitis. In adults, the case-fatality rate was higher than children at 9.7 per 100 cases (95% CI 8.8 to 10.7), 12.5 in herpes encephalitis (95% CI 10.4 to 14.3), and 8.5 per 100 cases without a diagnosis specified (95% CI 7.2 to 9.7). The mean length of stay for adults was 22.4 days, which increased to 30 days for those with herpes or “other” diagnoses compared with 17 days for cases without a diagnosis specified.

**Table 3:** Hospitalizations of viral encephalitis, deaths, and duration of stay, England, April 1, 1989–March 31, 1998^a^

Diagnosis	No. of cases	No. of deaths (%)	Case-fatality rate/100 cases	95% CI	Length of stay in days	95% CI
Exotic	64	1 (0.2)	1.6	0.01 to 8.6	16.5	7.5 to 25.5
Herpes viruses						
Herpes simplex virus	1,419	141 (33.8)	10	8.4 to 11.6	26.6	23.6 to 29.6
VZV	333	25 (6.0)	7.6	4.9 to 10.9	11.8	10.0 to 13.4
Others						
Measles	86	1 (0.2)	1.2	0.01 to 6.3	6.8	3.1 to 10.5
Mumps	30	1 (0.2)	3.3	0.01 to 15.8	4.1	2.9 to 5.3
Rubella	26	2 (0.5)	7.7	0.9 to 25.1	76.0	0.0 to 174.0
LCMV	7	0 (0.0)	0		7.2	2.1 to 12.3
Adenoviruses	129	6 (1.4)	4.7	1.8 to 9.8	8.5	6.8 to 10.2
Other	480	33 (7.9)	6.8	4.7 to 9.4	18.8	12.4 to 25.1
Unspecified viral infection	3,840	207 (49.6)	5.5	4.7 to 6.2	14.8	13.7 to 15.9
Total	6,414	417 (100)	6.5	5.9 to 7.1	17.5	16.4 to 18.6

^a^CI, confidence interval; VZV, varicella-zoster virus 1; LCMV, Lymphocytic choriomeningitis virus.

### Evidence of Clustering

For nonspecific cases of viral encephalitis, the frequency of hospitalizations (noted by week) indicated that some clustering of undiagnosed viral encephalitis had occurred during the 9 years of study since the upper prediction limits at 99.5% and 99.95% were exceeded in 6 weeks. Three of the six clusters occurred closely together in 1993 (weeks 45, 47, and 51), which suggests an overall period of higher incidence during this time. The Poisson regression model suggested clustering of encephalitis of unknown cause in district health authorities, and the total number of cases occurring in a week was greater than the prediction limit at 99.95% in three districts. The model did not identify any clusters when the period was extended to those occurring within 1 month.

### Comparison of Data Sources

Significant underreporting of viral encephalitis was seen in the routine systems when compared to hospital episode statistics. A total of 215 cases of acute infectious encephalitis were identified through NOIDS between April 1, 1989, and March 31, 1998, compared to 6,414 identified in hospital episode statistics. This disparity implies that almost all (97%) hospitalized cases were not formally reported.

A total of 599 cases of viral encephalitis were identified through the laboratory reporting system from January 1, 1990, to December 31, 1998. Fifty nine per cent of these cases were herpes (86 herpes simplex virus 1, 48 herpes simplex virus 2, and 219 subtype not known), 21% varicella and 4% adenovirus. The remaining 16% of cases included 26 cytomegalovirus, 15 Epstein-Barr virus, 20 measles virus, and 13 mumps. Of herpes encephalitis, 19% (66/353) of cases occurred in children and 4.8% (17/353) in neonates. Of herpes encephalitis cases in neonates that were typed, seven were herpes simplex virus 2 and two were herpes simplex virus 1.

The number of laboratory reports of viral encephalitis increased from 52 in 1990 to 80 in 1991, falling to 55 in 1993, and rising again to a peak of 104 in 1998. Excluding those cases for which no specific ICD-9 or ICD-10 code exists and which could not have been identified in hospital episode statistics, 558 cases of viral encephalitis were reported through the laboratory system from January 1, 1990, to March 31, 1998. This total represents approximately 71% underreporting when compared to 1,867 hospitalizations of viral encephalitis with a specific infection reported (Table 4). This underreporting varied by virus, ranging between 28% and 100%.

**Table 4:** A comparison of cases and deaths attributed to viral encephalitis with a specific diagnosis identified through hospital episode statistics, the laboratory reporting system, and the Office of National Statistics, England^a^

Diagnosis	Cases			Deaths		
	HES^b^	Laboratory reports^b^	Estimate of underreporting in laboratory reports (%)	HES^c^	ONS^c^	Estimate of underreporting in laboratory reports (%)
Herpes	1,308	353	73	85	104	22
VZV	325	124	62	24	34	42
Measles	71	43	39	0	1	0
Mumps	18	13	28	2	0	100
Rubella	23	1	96	0	0	0
LCMV	7	0	100	0	0	0
Adenoviruses	115	24	79	1	0	100
Total	1,867	558	70	112	139	24

^a^HES, hospital episode statistics; Herpes, herpes simplex virus; VZV, varicella-zoster virus 1; LCMV, Lymphocytic choriomeningitis virus.

^b^January 1, 1990–March 31, 1998.

^c^January 1, 1993–March 31, 1998.

Hospital episode statistics showed an underascertainment of deaths. Viral encephalitis was given as the final underlying cause of 622 deaths certified from January 1, 1993, to March 31, 1998. In that comparative period, hospital episode statistics showed 259 deaths, only 42% of those formally certified and reported to the Office of National Statistics. Most (483/622) certified deaths from viral encephalitis did not have a specific diagnosis; however, 139 deaths did. We compared the 139 deaths to the 112 identified in hospital episode statistics for which a specific diagnosis was recorded (Table 4).

## Discussion

The public health impact of viral encephalitis in England cannot be clearly defined with existing data sources. During our study of viral encephalitis in England, we found that more cases of viral encephalitis were identified in hospital episode statistics than in routine surveillance systems. Most cases requiring hospitalization are viral encephalitis with unknown etiology; of those cases with a specific diagnosis recorded, herpes is the most common cause. Adults account for the majority of case-patients, and rates have increased in those aged >65 years. Our analysis of cases of viral encephalitis without a specific diagnosis in hospital episode statistics identified clusters that would have otherwise gone undetected. Timely hospital episode statistics would provide a more comprehensive surveillance than any existing system.

The coding system used for the hospital diagnoses recorded in hospital episode statistics changed from ICD-9 to ICD-10 beginning on April 1, 1995. In some cases, diagnoses became less specific and in some cases more specific (e.g., herpes simplex virus was coded to herpes while VZV encephalitis had a specific ICD-10 code from 1995). In this study, we made some effort to compensate by including hospitalized cases with a diagnosis of chickenpox and an unspecified viral encephalitis infection. The change in coding did not appear to cause any major changes in the total number of hospitalized viral encephalitis cases.

Why are so few diagnoses in hospital episode statistics specific? Whether an appropriate investigation has been carried out, the extent to which a specific diagnosis is sought, and the quality of medical records affect the recording of specific diagnoses in hospital episode statistics. Appropriate investigation is limited by the diagnostic techniques available; virus isolation from cerebrospinal fluid or the brain can delay a diagnosis because culturing is slow, sensitivity may be poor, and obtaining specimens may require special techniques. Determining the virus causing the encephalitis may not be seen as essential if all patients diagnosed with viral encephalitis are routinely given acyclovir, regardless of the virologic diagnosis. Regional variation in the proportion of hospitalizations without a specific diagnosis suggests that local laboratory practice or investigation by clinicians may differ. A nonspecific diagnosis is recorded in hospital episode statistics if the laboratory results arrive after discharge. During the period of study, the rapid and highly sensitive PCR test was not widely used in England; however, as the test becomes more widely available, the proportion of cases with a specific diagnosis in hospital episode statistics may increase. In this study, we found that adults with a specific diagnosis were older than those without a diagnosis, which is likely to reflect the age distribution of herpes, VZV, and other specific viral encephalitis diagnoses. A lower case-fatality rate, however, and mean length of stay in hospital suggests that these infections without a specific diagnosis are less severe.

With the emergence of new diseases and availability of vaccines for some viruses, determining the cause of these infections is increasingly important (*19*,*21*–*25*). Information on etiology would also increase the specificity of cluster detection. In the past, routine data sources have been used alongside hospital episode statistics to determine the likely cause of hospitalizations without a specific diagnosis (*26*). These methods depend on seasonality of the viruses and are thus inappropriate for encephalitis.

To minimize overestimating the public health cost of viral encephalitis, multiple consultant episodes of care for a patient were counted as a single infection if <1 month elapsed between each. Hospital episode statistics do not include strict case definitions, which could also lead to overestimating. For example, between January 1, 1990, and March 31, 1998, a total of 71 cases of measles were reported in hospital episode statistics, most in children <5 years of age. These cases are unlikely to be true measles encephalitis since measles was rare in the 1990s following the introduction of the combined measles, mumps, and rubella vaccine in 1988 (*27*). Furthermore, none of the laboratory reports of measles encephalitis in this study were in infants, and only three were in children (aged 7, 11, and 14 years). The laboratory reports may also be overestimated. Cases have been included in the laboratory data based on a single high serum antibody titer. Had a stricter case definition been used, as has been suggested for measles (*28*), even fewer cases of viral encephalitis would have been identified in the laboratory system.

Hospital episode statistics identified a lower number of deaths from viral encephalitis than the number reported routinely to the Office of National Statistics. Deaths in hospital episode statistics are known to be incomplete since patients with encephalitis may die after discharge, and the death may be recorded in an episode that does not include encephalitis as a diagnosis and was thus missed in the data extraction (*29*).

Some epidemics of encephalitis in England are undetected in routine surveillance systems and undiagnosed in hospital episode statistics. This situation suggests the potential for another emerging infectious disease, such as West Nile infection, to occur in the U.K.; existing routine surveillance systems would be incapable of detecting this (*30*–*32*). Levels of disease in England are not as high as reported in a similar study in the United States (*33*); however, the proportion without a specific diagnosis is comparable. To improve surveillance, clinicians must be encouraged to report viral encephalitis, although notifications are not specific. Other existing surveillance systems, such as the British Pediatric Surveillance Unit (available from: URL: http://www.bpsu.inopsu.com/), could be modified to include a specific diagnosis of viral encephalitis. Alternatively, a sentinel surveillance system using existing laboratory networks and offering screening for a wide range of viruses could provide more accurate and timely data.
